# The Combined Inactivation of Intestinal and Hepatic ZIP14 Exacerbates Manganese Overload in Mice

**DOI:** 10.3390/ijms23126495

**Published:** 2022-06-10

**Authors:** Caitlin K. Fung, Ningning Zhao

**Affiliations:** School of Nutritional Sciences and Wellness, The University of Arizona, Tucson, AZ 85721, USA; caitlinfung@email.arizona.edu

**Keywords:** ZIP14, *SLC39A14*, manganese, intestine, liver, nutrition

## Abstract

ZIP14 is a newly identified manganese transporter with high levels of expression in the small intestine and the liver. Loss-of-function mutations in *ZIP14* can lead to systemic manganese overload, which primarily affects the central nervous system, causing neurological disorders. To elucidate the roles of intestinal ZIP14 and hepatic ZIP14 in maintaining systemic manganese homeostasis, we generated mice with single-tissue or two-tissue *Zip14* knockout, including intestine-specific (*Zip14*-In-KO), liver-specific (*Zip14*-L-KO), and double (intestine and liver) *Zip14*-knockout (*Zip14*-DKO) mice. *Zip14*^flox/flox^ mice were used as the control. Tissue manganese contents in these mice were compared using inductively coupled plasma mass spectrometry (ICP-MS) analysis. We discovered that although the deletion of intestinal ZIP14 only moderately increased systemic manganese loading, the deletion of both intestinal and hepatic ZIP14 greatly exacerbated the body’s manganese burden. Our results provide new knowledge to further the understanding of manganese metabolism, and offer important insights into the mechanisms underlying systemic manganese overload caused by the loss of ZIP14.

## 1. Introduction

Manganese is an essential nutrient. Manganese deficiency leads to impaired growth, osteoporosis, dyslipidemia, asthma, and cognitive defects. Excess manganese in the body is toxic, and produces a spectrum of neurological and behavioral defects, clinically known as manganism. Therefore, manganese balance in the body needs to be tightly controlled to prevent its inadequacy or overload. Two organs, namely the intestine and the liver, play important roles in this control [1,2,3,4]. The intestine serves as the site for dietary manganese absorption [5,6,7]. After being absorbed into the blood circulation, manganese travels through the portal vein and is delivered to the liver where it can be stored, redistributed, or secreted as a bile conjugate for intestinal reabsorption or fecal excretion [1,8,9,10,11,12,13]. Manganese transporters expressed in the intestine and the liver are central to the regulation of systemic manganese metabolism.

ZIP14 (also known as solute carrier family 39 member 14, or SLC39A14) is a newly identified manganese transporter [14,15,16]. A genome-wide transcriptomics analysis of tissue samples from healthy individuals revealed that the greatest ZIP14 abundance is in the liver, followed by the small intestine—the two main organs involved in regulating systemic manganese metabolism [17]. Indeed, patients carrying loss-of-function mutations of *ZIP14* developed manganese toxicity and early-onset dystonia due to manganese hyperaccumulation in the brain [18,19,20,21,22]. Consistent with the human phenotype associated with ZIP14 deficiency, mice with whole-body *Zip14* knockout (*Zip14*^−/−^) exhibited manganese loading in the blood and the brain that were over 10 times the normal level [23,24,25], further indicating the indispensable role of ZIP14 in maintaining systemic manganese homeostasis.

Based on the observations that patients with a ZIP14 deficiency lacked hepatic manganese deposition and that *Zip14*^−/−^ mice had reduced liver manganese, it was initially proposed that the inactivation of ZIP14 primarily impaired manganese delivery to the liver and subsequent clearance through biliary excretion, which in turn caused manganese accumulation in other organs including the brain [18]. However, liver-specific *Zip14*-knockout (*Zip14*-L-KO) mice did not display manganese hyperaccumulation in the blood or brain, despite significantly reduced liver manganese [25,26], in contrast with the results in *Zip14*^−/−^ mice.

In addition to the liver, the intestine also expresses high levels of ZIP14. Recent studies using intestine-specific *Zip14*-knockout (*Zip14*-In-KO) mice demonstrated a critical role for intestinal ZIP14 in controlling systemic manganese homeostasis because *Zip14*-In-KO mice developed increased manganese in both the blood and the brain [26,27]. However, the brain manganese loading observed in *Zip14*-In-KO mice was much less severe when compared with that seen in *Zip14*^−/−^ mice of a similar age [26,28]. We hypothesized that, since hepatic ZIP14 was intact in *Zip14*-In-KO mice, excess manganese resulting from increased manganese absorption could be partially removed through hepatobiliary excretion, leading to less severe manganese loading in *Zip14*-In-KO mice. Therefore, the present study aimed to further elucidate the mechanism of systemic manganese homeostatic regulation by examining manganese levels in mice with ZIP14 knockout in both the intestine and the liver (*Zip14* double knockout, or *Zip14*-DKO).

## 2. Materials and Methods

### 2.1. Animals, Genotyping and Tissue Collection

All procedures for animal experiments were approved by the Institutional Animal Care and Use Committees (IACUC) of the University of Arizona (Protocol number: 16–172). Animal cages containing less than 5 mice were kept at 21–22 °C with 12 h light/dark cycles. Mice were provided with tap water *ad libitum*, and fed a traditional rodent diet (Teklad 7913; Envigo, Indianapolis, IN, USA). Mice carrying the *Zip14* conditional allele (*Zip14*^flox/flox^) have been described previously [26]. Mice expressing Cre recombinase under the intestine-specific (Vil-Cre) or liver-specific (Alb-Cre) promoter were purchased from the Jackson Laboratory (Bar Harbor, ME, USA). *Zip14*^flox/flox^ mice were bred with Vil-Cre or Alb-Cre mice to obtain *Zip14*^flox/−^Vil-Cre^+^ or *Zip14*^flox/−^Alb-Cre^+^ mice. *Zip14*^flox/−^Vil-Cre^+^ and *Zip14*^flox/−^Alb-Cre^+^ mice were then crossbred to produce *Zip14*^flox/−^Vil-Cre^+^Alb-Cre^+^ mice. To generate tissue-specific *Zip14* knockout and the control littermates, *Zip14*^flox/−^Vil-Cre^+^Alb-Cre^+^ mice were bred to produce all four mouse stains used in this study, including *Zip14*^flox/flox^ (control), *Zip14*^flox/flox^Vil-Cre^+^ (*Zip14*-In-KO), *Zip14*^flox/flox^Alb-Cre^+^ (*Zip14*-L-KO), and *Zip14*^flox/flox^Vil-Cre^+^Alb-Cre^+^ (*Zip14*-DKO). A Mouse Direct PCR kit (Bimake, Houston, TX, USA) and the following primers were used to determine animal genotypes. For *Zip14*^flox/flox^ mice: Forward, 5′-GAT TCC TCC AAA GTC AAG TAG AGC G-3′; Reverse, 5′-GCT TGG AAG GGC TGG GTG CA-3′. Genotyping procedures for Vil-Cre and Alb-Cre mice were performed according to the company’s instructions. All mice were sacrificed at 9 weeks of age after anesthesia with ketamine/xylazine. Blood samples were collected via cardiac puncture. Mouse tissues were immediately frozen in liquid nitrogen after collection and stored in a −80 °C freezer for further analyses.

### 2.2. Metal Content Measurement

The metal contents of mouse tissues were analyzed by inductively coupled plasma mass spectrometry (ICP-MS) at the Arizona Laboratory for Emerging Contaminants (ALEC). Frozen tissues were weighed and dried in an oven at 80 °C for at least 2 days until they reached a constant weight. Dried tissues were digested with 2 mL of 70% concentrated HNO_3_ at room temperature (RT) overnight, and then at 80 °C for 6 h, followed by 60 °C overnight. Tissue digests (400 μL) were diluted using 9 mL of Milli-Q water to a total volume of 9.4 mL at a final concentration of 3% HNO_3_. For blood samples, 50 μL of blood was digested in 280 μL of 70% HNO_3_, incubated overnight at RT, and then at 80 °C for 4 h. The digested samples (200 μL) were diluted in 3.8 mL of Milli-Q water to a total volume of 4 mL at a final concentration of 3% HNO_3_. The metal contents were analyzed using the Agilent 7700 × ICP-MS instrument (Agilent Technologies, Santa Clara, CA, USA) at the ALEC. The protocol for analytical QA/QC was adapted from US EPA Method 200.8 for ICP-MS analysis. A multi-element stock solution (SPEX CertiPrep, Metuchen, NJ, USA) was used to prepare calibration standards, including at least 7 points with correlation coefficients higher than 0.995. The QC protocol included a continuing calibration blank, a continuing calibration verification solution, and at least one quality control sample to be analyzed after calibration, after 12 samples and at the completion of all sample analyses. Acceptable QC responses were between 90% and 110% of the certified values.

### 2.3. Western Blot Analysis

Mouse tissues were lysed in NETT buffer (150 mM NaCl, 5 mM EDTA, 10 mM Tris, 1% Triton X-100, and 1× Protease inhibitor cocktail). Protein concentrations were determined using the RC DC protein assay (Bio-Rad Life Science, Hercules, CA, USA). Equal amounts of protein were mixed with Laemmli buffer and incubated at 37 °C for 30 min. Proteins were electrophoretically separated on 10% sodium dodecyl sulfate polyacrylamide gels and transferred to nitrocellulose membranes (GVS, Sanford, ME, USA). After 1 h incubation with blocking buffer (5% non-fat dry milk in TBST (10 mM Tris/HCl, 150 mM NaCl, 0.1% 1 mL Tween-20, pH 7.5)) at RT, membranes were incubated with rabbit anti-mZIP14 antibody (1:1000) overnight at 4 °C. The generation of anti-mZIP14 antibody was described previously [26]. After incubation with the anti-mZIP14 antibody, nitrocellulose membranes were then washed 4 times with TBST (5 min/wash), and incubated for 1 h at RT with the donkey anti-rabbit horseradish peroxidase (HRP)-conjugated secondary antibody (1:5000) (GE healthcare, Chicago, IL, USA). Before imaging, membranes were washed two times (5 min/wash) with TBST, followed by two washes with TBS (5 min/wash) prior to imaging using the ChemiDoc MP system and Image Lab software (Bio-Rad). To confirm equivalent loading, the membranes were stripped for 15 min in Restore PLUS Western blotting stripping buffer (Thermo Fisher Scientific, Waltham, MA, USA), blocked for 1 h in blocking buffer, and reprobed with HRP-conjugated antibodies against GAPDH (1:20,000) or beta-ACTIN (1:20,000, Proteintech, Rosemont, IL, USA).

### 2.4. Statistical Analysis

Data were expressed as mean ± standard deviation of the mean (SD). Data homogeneity of variance was determined using the Brown–Forsythe test for each dataset, and no significant differences in the SDs were found. Comparisons between groups in each dataset were performed by one-way analysis of variance (ANOVA) followed by Bonferroni’s post-hoc tests using PRISM 8 software (GraphPad, La Jolla, CA, USA). For the post-hoc comparisons, a *p*-value < 0.05 was considered statistically significant with: * *p* < 0.05; ** *p* < 0.01; *** *p* < 0.001 and **** *p* < 0.0001.

## 3. Results

### 3.1. Generation of Mice with Tissue-Specific Zip14 Deletion

**Figure 1:**
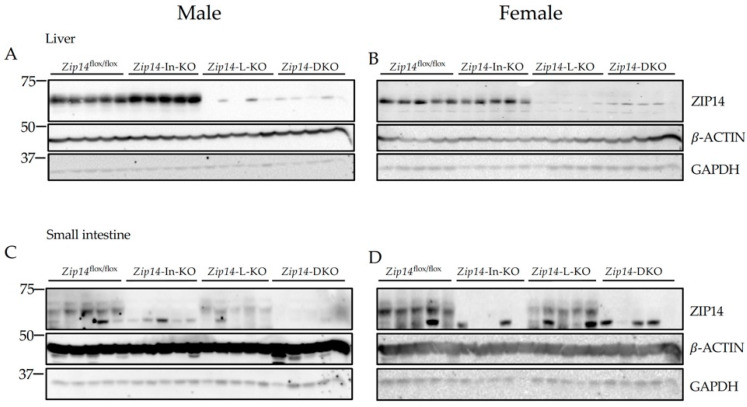
Western blot analyses to confirm tissue-specific *Zip14* knockout in mice. *Zip14*^flox/flox^ mice were bred with tissue-specific Cre transgenic mice to generate mice with *Zip14* knockout in the intestine, the liver, or in both tissues. Mice were sacrificed and tissues were collected at 9 weeks of age. Western blot images of (**A**,**B**) the liver and (**C**,**D**) small intestine samples from control (*Zip14*^flox/flox^), intestine-specific *Zip14* KO (*Zip14*-In-KO), liver-specific *Zip14* KO (*Zip14*-L-KO), and (intestine and liver)-specific *Zip14* KO (*Zip14*-DKO) mice (**A**,**C**: male mice; **B**,**D**: female mice, *n* = 5). Both *β*-ACTIN and GAPDH were used as loading controls.

To determine the coordinated regulation of manganese metabolism by the intestine and the liver, we generated a double-tissue *Zip14*-knockout mouse model (*Zip14-*DKO) with the inactivation of *Zip14* in both the intestine and the liver, then collected animal tissues at 9 weeks of age. For comparison with the age-matched single-tissue *Zip14* knockout, we also generated mice with a *Zip14* deletion in the intestine or the liver (*Zip14*-In-KO or *Zip14*-L-KO mice). Western blot analyses of the liver (Figure 1A,B) and the intestine tissue samples (Figure 1C,D) confirmed that the ZIP14 protein was indeed absent in the corresponding sites of relevant single- or double-tissue-specific knockout mice.

**Figure 2:**
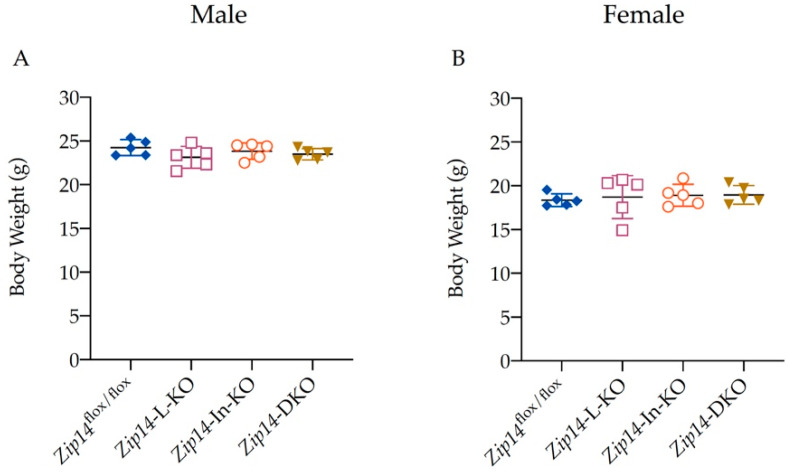
Deletion of ZIP14 in the intestine and/or liver did not affect the body weight in mice. Body weights of the 9-week-old control, *Zip14*-In-KO, *Zip14*-L-KO, and *Zip14*-DKO mice were plotted. (**A**) Male mice (*n* = 5). (**B**) Female mice (*n* = 5). Data were expressed as mean ± standard deviation (SD). Statistical analysis was performed using one-way analysis of variance (ANOVA) followed by the Bonferroni post-hoc test. No significant differences were found between these groups.

Reduced body weight was found to be a common phenotype of gene knockout in mice [29]. A previous report has found that with whole-body *Zip14* knockout, *Zip14*^−/−^ mice developed growth retardation, shorter body length, and lower body weight [30]. Therefore, we measured mouse body weights to determine the effect of tissue-specific *Zip14* deletion on animal growth. We found that body weight did not differ between the four animal genotypes examined in this study, (Figure 2A,B), indicating that mice grew at a similar rate between these groups.

### 3.2. Blood Manganese Increases Significantly in Zip14-DKO Mice

**Figure 3:**
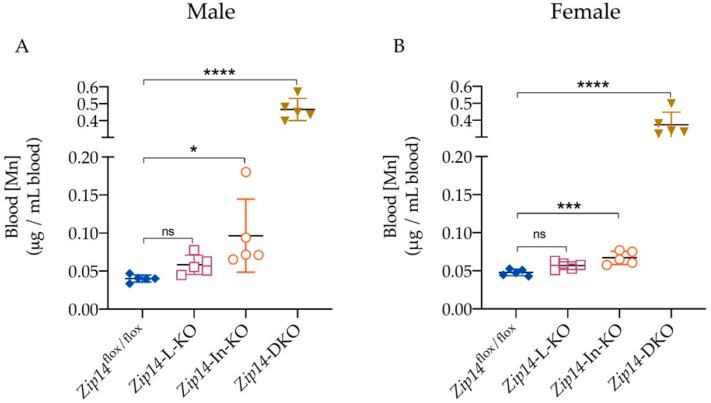
Blood manganese increased significantly in *Zip14*-DKO mice. Blood manganese (Mn) concentrations were measured by inductively coupled plasma mass spectrometry (ICP-MS) for (**A**) male mice (*n* = 5) and (**B**) female mice (*n* = 5) at 9 weeks of age. Data were expressed as mean ± SD and were subjected to a one-way ANOVA followed by the Bonferroni post-hoc test to compare the mean of each knockout group to the control group. * *p* < 0.05, *** *p* < 0.001 and **** *p* < 0.0001. “ns” indicates no significant differences.

Since whole-blood manganese content can be used as an indicator for body manganese status in rodents [31], we first measured manganese content in the blood using ICP-MS analysis. Consistent with previous reports [25,26], we found that there was no significant difference in blood manganese between the 9-week-old control and *Zip14*-L-KO mice, suggesting that the hepatic ZIP14 does not serve as a primary regulator for systemic manganese homeostasis under normal conditions (Figure 3A,B). In contrast, the blood manganese in the 9-week-old *Zip14*-In-KO mice was about 2.4 times and 1.4 times as high as the control values for male and female mice, respectively (0.0402 ± 0.0047 μg/mL vs. 0.0965 ± 0.0480 μg/mL for male mice; 0.0477 ± 0.0042 μg/mL vs. 0.0671 ± 0.0087 μg/mL for female mice), indicating that the deletion of intestinal ZIP14 increased the body’s manganese burden (Figure 3A,B).

However, the extent of manganese accumulation caused by the loss of intestinal ZIP14 was not as severe as that observed in *Zip14*^−/−^ mice because it has been reported that the blood manganese levels in *Zip14*^−/−^ mice of a similar age were about 15 times as high as the wild-type (WT) littermates [28]. We hypothesize that since hepatic ZIP14 was intact in *Zip14*-In-KO mice, excess manganese resulting from the loss of intestinal ZIP14 could be partially removed through hepatobiliary excretion, leading to less severe manganese loading in *Zip14*-In-KO mice. Indeed, with the deletion of both intestinal and hepatic ZIP14, *Zip14-*DKO mice had a blood manganese concentration about 12 times (0.0402 ± 0.0047 μg/mL vs. 0.4666 ± 0.0652 μg/mL for male mice) or 8 times (0.0477 ± 0.0042 μg/mL vs. 0.3734 ± 0.0740 μg/mL for female mice) as high as the value of sex-matched littermate controls (Figure 3A,B), indicating that both intestinal and hepatic ZIP14 contributed to the regulation of systemic manganese homeostasis.

**Figure 4:**
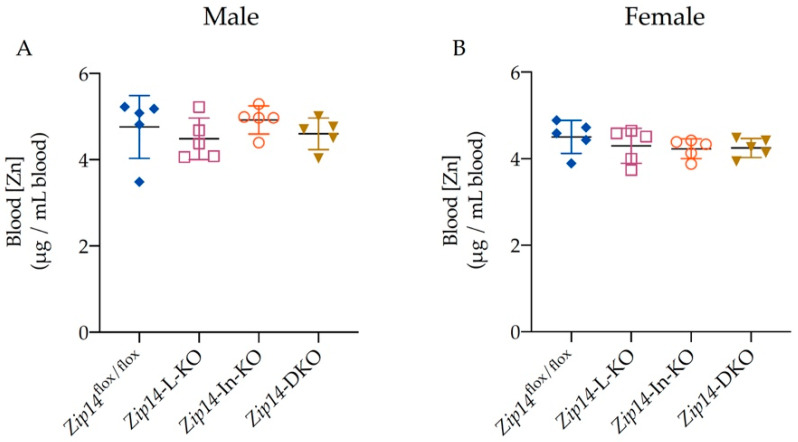
Tissue-specific *Zip14* knockout did not alter blood zinc levels. Blood zinc (Zn) concentrations were measured by ICP-MS for (**A**) male (*n* = 5) and (**B**) female mice (*n* = 5). Data were expressed as mean ± SD and statistical analysis was performed using one-way ANOVA followed by the Bonferroni post-hoc test. No significant difference was observed between each group of *Zip14* knockout and control mice.

As a divalent metal transporter, ZIP14 can mediate the transport of a variety of essential trace metals including zinc [14]. ICP-MS measurements demonstrated that, in contrast to the significantly altered blood manganese, blood zinc levels did not differ between the four groups of mice examined in this study (Figure 4A,B), suggesting that loss of ZIP14 in the intestine and/or in the liver did not significantly affect the body’s zinc metabolism.

### 3.3. Liver Manganese Increases in Zip14-In-KO Mice, but Decreases Significantly in Both Zip14-L-KO and Zip14-DKO Mice

**Figure 5:**
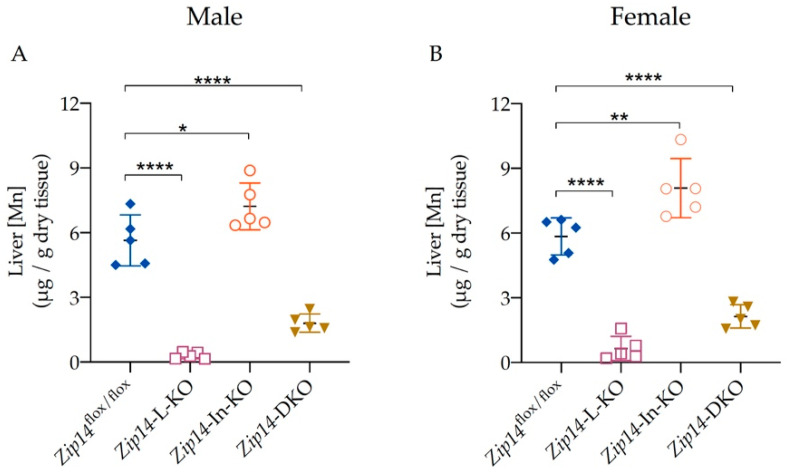
Liver manganese increased in *Zip14*-In-KO mice but decreased in both *Zip14*-L-KO and *Zip14*-DKO mice. Liver Mn contents were measured by ICP-MS in (**A**) male (*n* = 5) and (**B**) female mice (*n* = 5). Data were expressed as mean ± SD and were subjected to a one-way ANOVA followed by the Bonferroni post-hoc test to compare the mean of each knockout group to the control group. * *p* < 0.05, ** *p* < 0.01 and **** *p* < 0.0001.

To further determine the effect of tissue-specific *Zip14* knockout, we next analyzed liver manganese. The 9-week-old *Zip14*-L-KO mice had about 90–95% decreased manganese content in the liver when compared with the sex-matched *Zip14*^flox/flox^ control animals (Figure 5A,B) (5.648 ± 1.18 μg/g vs. 0.3069 ± 0.1494 μg/g for male mice; 5.843 ± 0.8622 μg/g vs. 0.6499 ± 0.2523 μg/g for female mice). These results support the notion that ZIP14 is the major transporter mediating manganese uptake into the liver [24,25,26]. In contrast, for *Zip14*-In-KO mice, a significant increase in liver manganese (increased by 28% in male mice and by 38% in female mice) was evident (Figure 5A,B), reinforcing the idea that the deletion of intestinal ZIP14 increases the body’s manganese burden. Similar to *Zip14*-L-KO mice, *Zip14-*DKO mice displayed reduced liver manganese when compared with *Zip14*^flox/flox^ control mice, indicating that the hepatic manganese accumulation was impaired in both *Zip14*-L-KO and *Zip14-*DKO mice.

### 3.4. ZIP14 Deletion in Both the Liver and Intestine Significantly Increases Brain Manganese Accumulation

**Figure 6:**
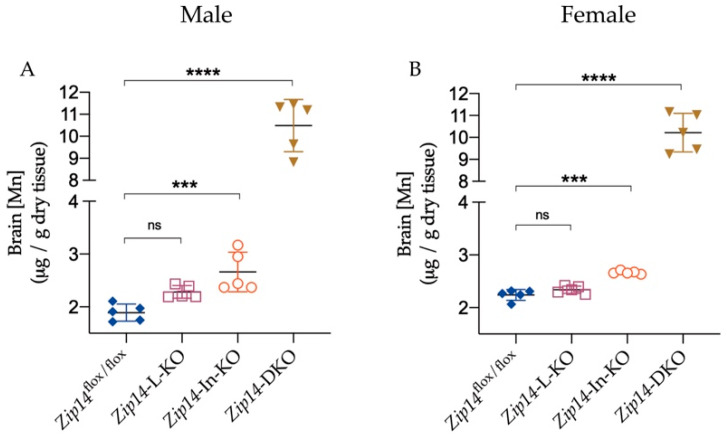
Combined inactivation of intestinal and hepatic ZIP14 greatly exacerbated brain manganese accumulation. Brain Mn concentrations were measured by ICP-MS in (**A**) male (*n* = 5) or (**B**) female mice (*n* = 5). Data were expressed as mean ± SD and were subjected to a one-way ANOVA followed by the Bonferroni post-hoc test to compare the mean of each knockout group to the control group. *** *p* < 0.001 and **** *p* < 0.0001. “ns” indicates no significant differences.

ZIP14 inactivation primarily impairs the central nervous system and leads to neurodegenerative symptoms due to manganese hyperaccumulation in the brain. For example, the brain manganese in 6-to-12-week-old *Zip14*^−/−^ mice was reported to be about 10 times as high as that in WT mice [28]. In the present study, we examined the effect of tissue-specific *Zip14* deletion on brain manganese in 9-week-old mice and found that *Zip14*-L-KO mice had normal brain manganese while *Zip14*-In-KO mice developed increased brain manganese loading (increased by 40% in male mice and by 20% in female mice) when compared with the *Zip14*^flox/flox^ control mice (Figure 6A,B). These results further demonstrated an increased systemic manganese burden with the loss of intestinal ZIP14. Importantly, with the deletion of ZIP14 in both the intestine and the liver, *Zip14-*DKO mice displayed exacerbated manganese loading in the brain (5.5 times and 4.6 times as high as the controls for male and female mice, respectively), demonstrating that the liver works in tandem with the intestine to regulate systemic manganese homeostasis.

## 4. Discussion

Manganese metabolism needs to be tightly regulated to prevent adverse effects caused by an excess or a deficiency of this trace nutrient. Intestinal absorption and hepatobiliary excretion of manganese are two important physiological processes for the regulation of systemic manganese homeostasis [32,33,34,35]. However, mechanisms underlying such regulation are not well understood. ZIP14 is a newly identified manganese importer with high expression levels in both the small intestine and the liver. Patients with loss-of-function mutations in *ZIP14* developed severe childhood-onset neurological disorders due to manganese accumulation in the brain; similarly, mice with whole-body *Zip14* knockout displayed manganese loading in the blood and brain at over 10 times the normal level, indicating an indispensable role for ZIP14 in maintaining systemic manganese homeostasis [18,19,21,22,23,24,25].

When the initial cases of *ZIP14* mutations in patients or *Zip14* knockout in mice were reported, it was thought that manganese hyperaccumulation caused by ZIP14 loss was primarily due to impaired manganese delivery to the liver, which in turn decreased manganese excretion through the bile, and caused manganese accumulation in the blood and other tissues, such as the brain. However, with hepatic ZIP14 inactivation, *Zip14*-L-KO mice had reduced liver manganese without developing manganese overload in other organs, indicating that hepatic ZIP14 was not the primary control for systemic manganese homeostasis under normal physiological conditions [25,26]. Then, a question arose naturally: How does hepatic ZIP14 contribute to the maintenance of systemic manganese homeostasis? In contrast to the results obtained from *Zip14*-L-KO mice, with intestinal ZIP14 inactivation, *Zip14*-In-KO mice displayed an increased manganese loading in both the liver and the brain, demonstrating an essential role for intestinal ZIP14 in regulating whole-body manganese metabolism [26,27]. However, the extent of brain manganese loading observed in *Zip14*-In-KO mice was rather mild when compared with that in *Zip14*^−/−^ mice. These results opened up a new question: Could the relatively mild degree of manganese loading in *Zip14*-In-KO mice be ascribed to the presence of hepatic ZIP14, which may facilitate the removal of excess manganese through the route of hepatobiliary excretion?

The present study created *Zip14*-DKO mice with *Zip14* inactivation in both the intestine and the liver to further reveal the mechanisms underlying ZIP14′s function in manganese metabolism. Age- and sex-matched *Zip14*-L-KO and *Zip14*-In-KO mice were also analyzed for comparison. Our results clearly showed that the deletion of both intestinal and hepatic ZIP14 exacerbated manganese loading in mice when compared with either single-tissue knockout (Figure 3, Figure 5 and Figure 6). Our findings indicate that, while the intestinal ZIP14 serves as a primary control for manganese metabolism, the hepatic ZIP14 is also essential for the maintenance of systemic manganese homeostasis under normal physiological conditions. The intestine and the liver are central for the regulation of nutrient metabolism. Our study demonstrated that combined defects in these two organs account for the onset of dysregulated manganese homeostasis seen in individuals lacking ZIP14.

In summary, the present study demonstrated, for the first time, the coordinated regulation of systemic manganese homeostasis by both intestinal and hepatic ZIP14, and enhanced our understanding of the disease mechanism underlying systemic manganese overload associated with ZIP14 loss.

## Data Availability

The data presented in this study are available on request from the corresponding author.
